# Large oculomotor schwannoma presenting as a parasellar mass: A case report and literature review

**DOI:** 10.4103/2152-7806.63910

**Published:** 2010-05-31

**Authors:** Sujit S. Prabhu, Janet M. Bruner

**Affiliations:** Department of Neurosurgery, The University of Texas M. D. Anderson Cancer Center, Houston, Texas, USA; 1 Department of Pathology, The University of Texas M. D. Anderson Cancer Center, Houston, Texas, USA

**Keywords:** Meningioma, oculomotor schwannoma, skull base

## Abstract

**Background:**

Large schwannomas arising from the oculomotor nerve are very rare. The common site of tumor occurrence in this nerve is the segment within the interpeduncular cistern and the cavernous sinus.

**Case description:**

We report a case of a large left-sided oculomotor nerve schwannoma with minimal clinical signs and symptoms of oculomotor nerve involvement resembling a large parasellar mass. The radiological features of the mass were more consistent with a medial sphenoid wing meningioma causing brain stem compression. Complete resection of the tumor was achieved via a left pterional approach. The patient developed complete third nerve palsy postoperatively.

**Conclusion:**

The management of these large benign tumors with brain stem compression includes surgical resection. Intraoperative anatomical preservation of the third nerve was impossible given its course in the tumor. We discuss the pertinent literature and management of large oculomotor schwannomas.

## INTRODUCTION

Schwannomas constitute about 7% of all intracranial tumors and commonly arise from the vestibulocochlear and trigeminal nerves. Motor nerve schwannomas arising from the oculomotor nerve are very rare. Approximately 30 cases of oculomotor nerve schwannomas have been described in the literature, of which 11 cases were described as large (≥2.5 cm). The management of these large tumors is especially challenging given the relationship of these tumors to the cranial nerves and the brainstem. Here we report a large oculomotor schwannoma and review the management of these uncommon tumors. 

### Case description

**Figure 1:**
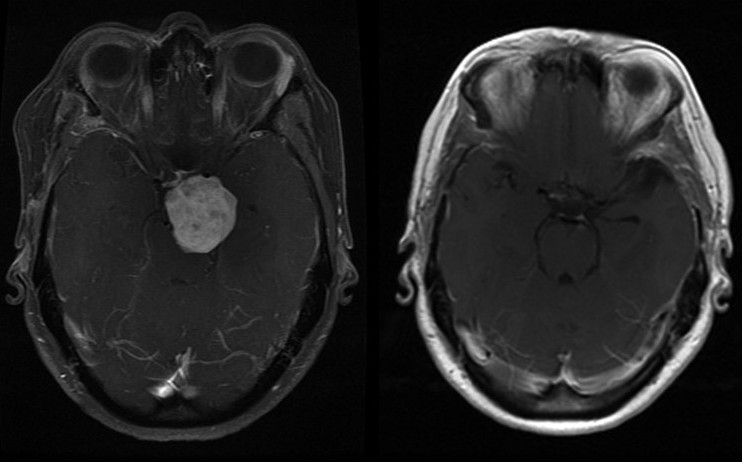
An axial gadolinium-enhanced contrasted T1-weighted MRI scan showing a large parasellar/ suprasellar mass with significant brainstem compression (a ). A postoperative axial gadolinium-enhanced T1-weighted MRI contrast scan 3 months later showing a complete resection of the mass with brainstem compression reversed (b)

**Figure 2:**
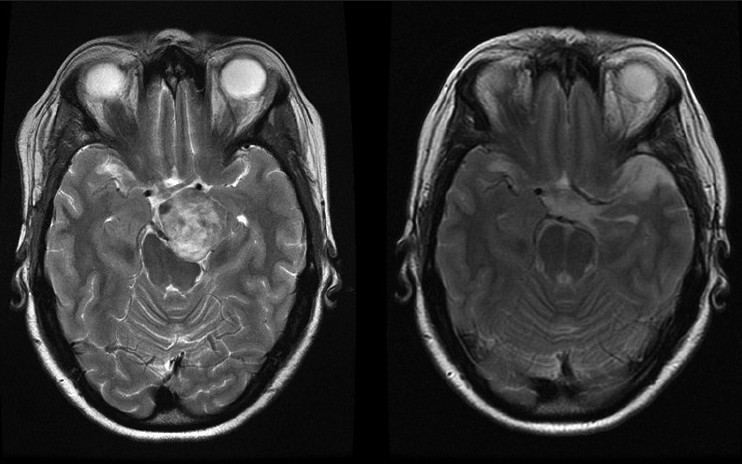
A preoperative (a) and a postoperative (b) T2-weighted axial MRI scan. The preoperative scan (a) shows a well-defined cerebrospinal fluid cleft around the mass

**Figure 3:**
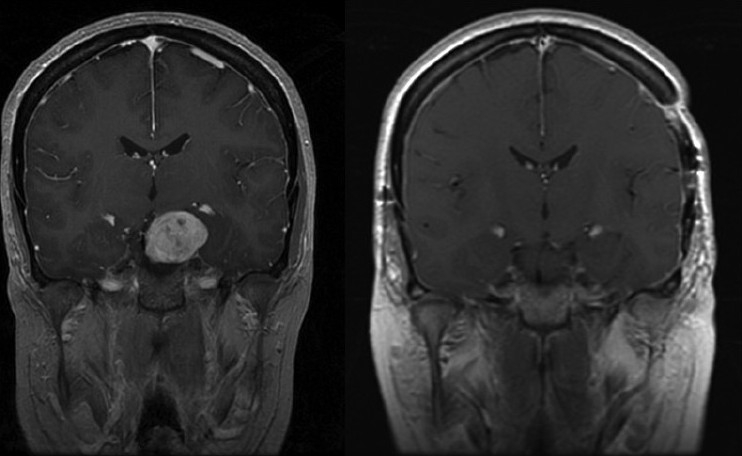
A coronal gadolinium-enhanced contrasted T1-weighted MRI scan showing a large parasellar/ suprasellar mass with significant brainstem compression (a). A postoperative coronal gadolinium-enhanced T1-weighted MRI contrast scan 3 months later showing a complete resection of the mass with brainstem compression reversed (b). A combined subtemporal and anterior Sylvian fissure-splitting approach was used to reach the tumor

A 38-year-old female developed symptoms of headache, dizziness, occasional diplopia and drooping of the left eyelid. Imaging confirmed a large left suprasellar mass (3.5 cm in diameter) with midbrain compression, suggesting a meningioma [Figures 1, 2 and 3]. A biopsy via a right pterional approach at an outside institution showed a benign spindle-cell neoplasm. The material was too small to definitively distinguish between meningioma and schwannoma. No tumor was resected at this operation. At MDACC her examination confirmed a bi-temporal hemianopsia related to previous surgery. There were no clinical signs of oculomotor palsy. The preoperative diagnosis in our patient was a meningioma, given the paucity of clinical findings. A left pterional craniotomy, drilling of the sphenoid wing and wide splitting of the Sylvian fissure for tumor access was carried out. The tumor was well encapsulated. After substantial debulking of the mass, the third nerve appeared from the interpeduncular cistern as a bundle and ran straight into the base of the mass. No other significant attachments of the tumor were recognized. Postoperatively, the patient developed complete third nerve paralysis on the left side. At 6-month follow-up, she continued to have complete third nerve palsy with no evidence of residual tumor [Figures 1, 2 and 3].

*Pathologic description:* Multiple rubbery fragments of tumor aggregated to a mass about 3.5 cm in diameter. Multiple histologic sections showed typical appearance of a benign schwannoma with short-spindled cells and random nuclear pleomorphism [Figure 4a]. Most of the tissue was the denser, more organized, Antoni A type, with very few Antoni B areas. No Verocay bodies were seen. Focal perivascular hemorrhage was identified. Occasional histiocytes and small aggregates of mature lymphocytes were sometimes present around blood vessels [Figure 4b]. A few blood vessels showed mural hyalinization. Immunohistochemistry for S-100 protein showed diffuse strong nuclear and cytoplasmic reactivity in the schwannoma [Figure 4c]. Because of the consideration of meningioma, an immunohistochemical stain for epithelial membrane antigen was performed but was entirely nonreactive [Figure 4d].

**Figure 4a:**
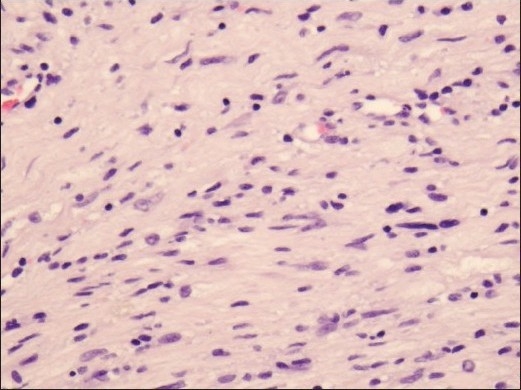
Spindle-cell tumor with hyalinized blood vessel walls (arrow) and small aggregates of mature lymphocytes (circles). Hematoxylin and eosin, ×10

**Figure 4b:**
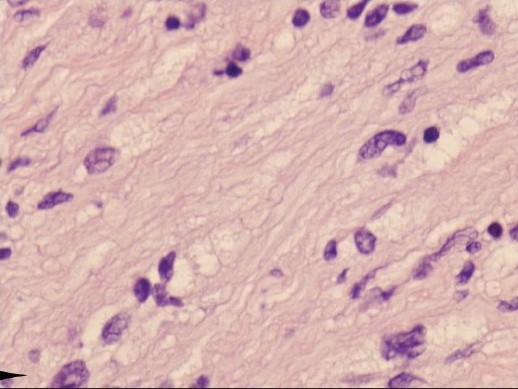
Scattered pleomorphic nuclei (arrows) in the schwannoma. Hematoxylin and eosin, ×20

**Figure 4c:**
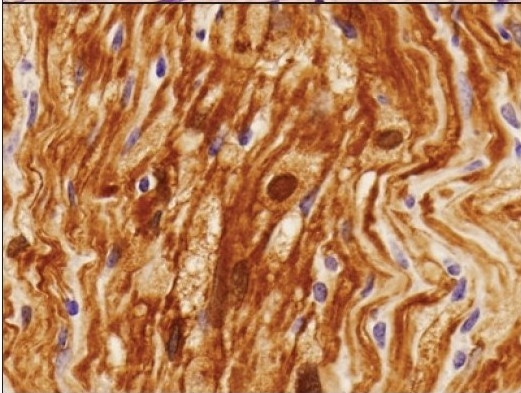
Strong diffuse immunoreactivity for S-100 protein in the schwannoma. S-100 protein immunohistochemistry, ×20

**Figure 4d:**
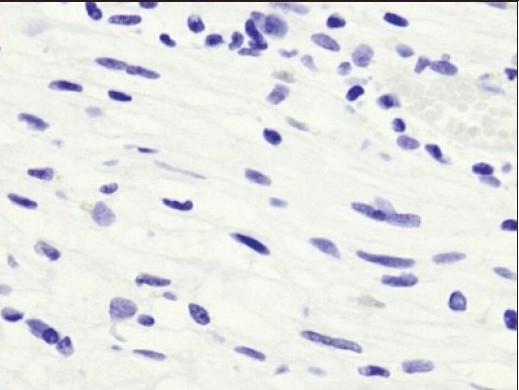
Immunohistochemistry for epithelial membrane antigen was negative in the schwannoma. Epithelial membrane antigen immunohistochemistry, ×20

## DISCUSSION

Schwannomas arising from the ocular nerves are extremely uncommon, unless associated with neurofibromatosis. Kovacs,[6] who in 1927 described an isolated oculomotor nerve schwannoma observed during an autopsy, was probably the first to report such a tumor. There are approximately 32 cases of well-documented oculomotor nerve schwannomas[15] in the literature, of which 11 cases described were large (2.5 cm or larger in diameter). These large tumors, because of their location, present unique diagnostic and surgical challenges to the neurosurgeon and will be the focus of this discussion. These large tumors typically present as a parasellar or suprasellar mass and can mimic a medial sphenoid wing or a posterior clinoidal meningioma. There are no radiological findings in these large parasellar tumors that can reliably distinguish schwannomas from meningiomas. Generally these large tumors are misdiagnosed as meningiomas preoperatively, given the paucity of clinical signs and symptoms of third cranial nerve involvement, as seen in our case [Table 1].

Of the 12 patients (including our patient), there were 9 females and 3 males with a median age of 41.5 years. Celli *et al.*[3] divided oculomotor cranial nerve schwannomas into three groups, i.e., 1) cisternal, 2) cisternocavernous and 3) cavernous lesions. This classification was based on the preferred extension of these tumors. All the cases described except 2 had a large cisternal extension based on imaging and operative findings. The explanation for this could be that the ventral cistern of the brainstem is a potential space where these slow-growing tumors expand and can also cause brainstem compression. Five (42%) patients presented with signs of hemiparesis as a result of brainstem compression. The cases described by Oakamoto *et al.*[12] and Barat *et al.*[1] had intraorbital extension along with a large parasellar component. In both these cases, exophthalmos was the presenting symptom. Ten patients presented with some ocular signs or symptoms, with 4 (33%) out of 12 patients presenting with a mild ptosis. The ocular findings surprisingly were minimal, given the large size of the tumors [Table 1]. Multiple cranial nerve deficits were seen in only 1 case described by Barat *et al.*[1] involving the fourth, fifth and sixth nerves. This tumor, however, had a large intracavernous component. 

Seven (58%) out of 12 patients underwent a complete resection[4,5,7,8,10,11] ; 4, a partial or subtotal resection[1,9,12,14] ; and in 1 patient, the resection status was not specified.[2] Of the 7 patients who underwent a complete resection, 6 (86%) patients had complete third nerve palsy following surgery. Niazi and Boggan[11] described a case where the tumor was resected completely after the second operation (4 years apart). The patient, however, only experienced incomplete third nerve palsy. 

**Table 1:** Summary of histologically verified large (≥2.5 cm) oculomotor schwannomas as described in the literature

Author, Year	Age (yrs), Sex	Preoperative symptoms/signs	Tumor size (cm)	Extent of resection	Location along third nerve	Postoperative third nerve deficits
Broggi and Franzini, 1981	45, M	Hemiparesis, central seventh nerve palsy	3^a^	NS	NS	NS
Hiscott and Symon, 1982	58, F	Headaches, drowsiness, hemiparesis, minimal ptosis	4^a^	Total	cisternal	Complete third nerve palsy
Luenda *et al*., 1982	11, M	Headaches, hemiparesis, impaired upward gaze	5.5^a^	Total	NS	Third nerve palsy
Oakamoto *et al*., 1985	52, F	Exophthalmos, convulsions, third nerve findings	4^a^	Subtotal	Parasellar, intraorbital	Unchanged
Lunardi *et al*., 1989	60, F	Headache, diplopia, hemiparesis	3.5^a^	Total	Cisternal	Complete third nerve palsy
Mehta *et al*., 1990	19, F	Cerebellar signs, minimal ptosis, anisocoria	5^a^	Subtotal	Parasellar, posterior fossa	Complete third nerve palsy
Takano *et al*., 1990	65, F	Diplopia, ptosis	2.5	Partial	Parasellar, middle fossa	Complete third nerve palsy
Barat *et al*., 1992	27, F	Exophthalmos, visual impairment, cranial nerves IV, V, VI paresis	4^a^	Subtotal	Intracavernous ophthalmic canal	Complete third nerve palsy
Niazi and Boggan, 1994	13, F	Ptosis, anisocoria, hemiparesis	3^a^	Two operations: 1985 (subtotal), 1989 (complete)	Parasellar, cavernous, cisternal	Incomplete third nerve palsy
Kachara *et al*., 1998	61, M	Headaches, diplopia, minimal ptosis, trochlear nerve paresis	5	Total	Parasellar, suprasellar	Complete third and fourth nerve palsy
Netuka and Benes, 2003	12, F	Headaches	2.8	Total	Parasellar, suprasellar, prepontine	Complete third nerve palsy improved at 12 months
Prabhu *et al*.,2009	38, F	Headaches, diplopia	3.5	Total	Parasellar, suprasellar, prepontine	Complete third nerve palsy

a Estimated based on findings from computed tomography or magnetic resonance imaging in the literature

Hiscott and Symon[4] describe a tumor with a large cisternal component, and at the end of the resection, describe the third nerve as "a stout bundle as it appeared from the interpeduncular cistern and ran straight into the mass, spread out and disappeared." We came across a finding similar to this in our patient, and third nerve could not be preserved and was sacrificed. Of the 4 patients who had an incomplete resection, 3 (75%) had a complete palsy of the third cranial nerve. Preserving function of the third nerve in these large tumors can be a challenge, as confirmed in this report, where 9 (75%) of the 12 patients operated (whether complete or partial resections) upon had complete third nerve palsy. In these large tumors, the distortion of the normal anatomy and intimate involvement of the nerve with the tumor make it difficult to preserve oculomotor nerve function after surgery. This would be an important message to be conveyed to patients who harbor these large tumors. 

Oculomotor nerve reconstruction may be important for cosmetic reasons (to avoid ptosis) and in cases involving blindness in the other eye. Nerve grafting, tried after resection of smaller tumors, produces only partial recovery.[13] None of the patients described in this report underwent any type of reconstructive procedure.

## CONCLUSION

Large schwannomas of the third cranial nerve are a rare entity and are frequently misdiagnosed as meningiomas on preoperative imaging because of the paucity of clinical signs and symptoms of cranial neuropathies. Although a significant number of these patients are left with complete third nerve palsy following surgical intervention, gross total resection of these tumors is recommended for treatment or prevention of brainstem compression. The patients need to be adequately counseled regarding the high morbidity from third nerve palsy associated with resection of these tumors.
